# Stable isotope metabolomics of pulmonary artery smooth muscle and endothelial cells in pulmonary hypertension and with TGF-beta treatment

**DOI:** 10.1038/s41598-019-57200-5

**Published:** 2020-01-15

**Authors:** Daniel Hernandez-Saavedra, Linda Sanders, Scott Freeman, Julie A. Reisz, Michael H. Lee, Claudia Mickael, Rahul Kumar, Biruk Kassa, Sue Gu, Angelo D’ Alessandro, Kurt R. Stenmark, Rubin M. Tuder, Brian B. Graham

**Affiliations:** 1 Department of Medicine, University of Colorado Anschutz Medical Campus, Aurora, CO USA; 2 Department of Biochemistry and Molecular Genetics, University of Colorado Anschutz Medical Campus, Aurora, CO USA; 3 Department of Medicine, University of California San Francisco, San Francisco, CA USA; 4 Department of Pediatrics, University of Colorado Anschutz Medical Campus, Aurora, CO USA

**Keywords:** Respiratory tract diseases, Translational research

## Abstract

Altered metabolism in pulmonary artery smooth muscle cells (PASMCs) and endothelial cells (PAECs) contributes to the pathology of pulmonary hypertension (PH), but changes in substrate uptake and how substrates are utilized have not been fully characterized. We hypothesized stable isotope metabolomics would identify increased glucose, glutamine and fatty acid uptake and utilization in human PASMCs and PAECs from PH versus control specimens, and that TGF-β treatment would phenocopy these metabolic changes. We used ^13^C-labeled glucose, glutamine or a long-chain fatty acid mixture added to cell culture media, and mass spectrometry-based metabolomics to detect and quantify ^13^C-labeled metabolites. We found PH PASMCs had increased glucose uptake and utilization by glycolysis and the pentose shunt, but no changes in glutamine or fatty acid uptake or utilization. Diseased PAECs had increased proximate glycolysis pathway intermediates, less pentose shunt flux, increased anaplerosis from glutamine, and decreased fatty acid β-oxidation. TGF-β treatment increased glycolysis in PASMCs, but did not recapitulate the PAEC disease phenotype. In TGF-β-treated PASMCs, glucose, glutamine and fatty acids all contributed carbons to the TCA cycle. In conclusion, PASMCs and PAECs collected from PH subjects have significant changes in metabolite uptake and utilization, partially recapitulated by TGF-β treatment.

## Introduction

Changes in cellular metabolism are increasingly recognized as a hallmark of pulmonary hypertension (PH) pathobiology^1–4^. Shifts in the uptake of metabolic substrates and how they are utilized downstream enables the disease phenotype of vascular cells in PH, including increased proliferation, apoptosis resistance, hypertrophy and vasoconstriction^3^. One critical metabolic shift observed in PH is an increase in glycolysis, which is thought to occur in resident vascular wall cells including pulmonary artery smooth muscle cells (PASMCs), endothelial cells (PAECs) and fibroblasts^5–7^. Increased glucose uptake can be demonstrated *in vivo* by increased uptake of the glucose analog ^18^F-fluorodeoxyglucose in the lung parenchyma of PH subjects^6,8^. The concept that glycolysis in PH is detrimental has led to investigation of the potential utility of dichloroacetate (DCA), which by blocking pyruvate dehydrogenase kinase causes increased glucose flux into the TCA cycle, and less glycolysis^9^. Glutamine uptake and metabolism by PAECs has also been shown to contribute to their disease phenotype^10^. However, comprehensive assessment of substrate uptake and how the substrates are utilized by pulmonary vascular cells in PH is lacking.

A potential driver of altered cellular metabolism is transforming growth factor β (TGF-β) signaling, which underlies many forms of heritable (through mutations in BMPR2 and other members of the TGF-β signaling superfamily) and idiopathic PAH, and PAH etiologies associated with other conditions such as autoimmune disease and schistosomiasis^11–13^. TGF-β induces cellular phenotypes which require energy and metabolic substrates, including proliferation, migration, contraction, and synthesis of cytokines and the extracellular matrix.

Here, we hypothesized that PASMCs and PAECs obtained from subjects with idiopathic pulmonary arterial hypertension (IPAH) will have increased glycolysis, glutaminolysis, and fatty acid β-oxidation compared to cells from control subjects. We assessed the metabolism of primary cells derived from diseased and human donor lungs using stable isotope metabolomics, an approach that allows assessment of uptake and downstream utilization of labeled substrates. We found evidence of increased glycolysis and pentose shunt flux particularly in PASMCs. We also found evidence of increased glutamine metabolism in PAECs but not PASMCs. Diseased PAECs had evidence of less fatty acid metabolism. We were able to phenocopy aspects of the altered metabolic phenotype by treating the cells with TGF-β, most notably the glycolytic shift in PASMCs. Overall, our results indicate PASMCs and PAECs in PH have quite different changes in their metabolic phenotype, and future therapeutic interventions targeting metabolism will likely benefit from cell compartment specificity.

## Results

We obtained primary PASMCs and PAECs from explanted IPAH and unsuccessful donor control lung specimens collected by the Pulmonary Hypertension Breakthrough Initiative (PHBI), a multi-center consortium that collects and distributes tissue specimens. We used N = 5 in each of the 4 categories (PASMCs and PAECs; diseased and control of each). The specimens had similar ages and sex distributions (Tables 1 and 2). Within these, we had N = 3 PASMCs and PAECs from the same diseased specimen, and N = 3 PASMCs and PAECs from the same control specimen. We added stable isotope-labeled metabolites–glucose, glutamine or a mixture of 4 long chain fatty acids–to the cell culture media, and following a 24 hour incubation, separated the supernatant from the cells and then performed metabolomics analysis on the samples.

**Table 1:** Age and sex of subjects for control PASMCs and PAECs.

Control Subjects			
PASMCs	PAECs	Age	Sex
X		25	M
X		55	F
X	X	24	M
X	X	50	F
X	X	36	F
	X	55	F
	X	49	M

**Table 2:** Age, sex, BMPR2 mutations identified, pre-transplant right heart catheterization data, and PAH medication usage of subjects for diseased PASMCs and PAECs. All diseased subjects were clinically diagnosed with idiopathic pulmonary arterial hypertension (IPAH).

Diseased Subjects							
Cells Used		Age	Sex	BMPR2 mut	Pre Txplt RHC		Medications
PASMCs	PAECs				mPAP	PVR^1^	PDE5inh/ ERA/Prost.^2^
					(mmHg)	(WU)	
X		57	F	N	82	27.3	S/A/E
X		16	M	N	63	20.5	T/-/E
X	X	40	M	N	73	16.8	S/A/T
X	X	27	F	c.76 + 5 G > A^3^	69	12.1	S/B/T
X	X	53	M	N	33	3.9	T/M/E
	X	16	F	N	95	N/A	S/-/T
	X	32	F	N	47	16.4	-/B/E

^1^Calculated using Fick cardiac output. N/A: data not available.

^2^PDE5inh: PDE5 inhibitor; ERA: Endothelin Receptor Antagonist; Prost.: Prostacyclin Analog.

Codes for each category (-: none used in that category):

PDE5 inhibitor: S: sildenafil, T: tadalafil

ERA: B: bosentan, A: ambrisentan, M: macitentan

Prost.: E: epoprostenol, T: treprostinil (intravenous).

^3^Mutation judged likely pathogenic as it may disrupt mRNA splicing.

### Increased glucose uptake and utilization by glycolysis and the pentose shunt in diseased PASMCs

**Figure 1:**
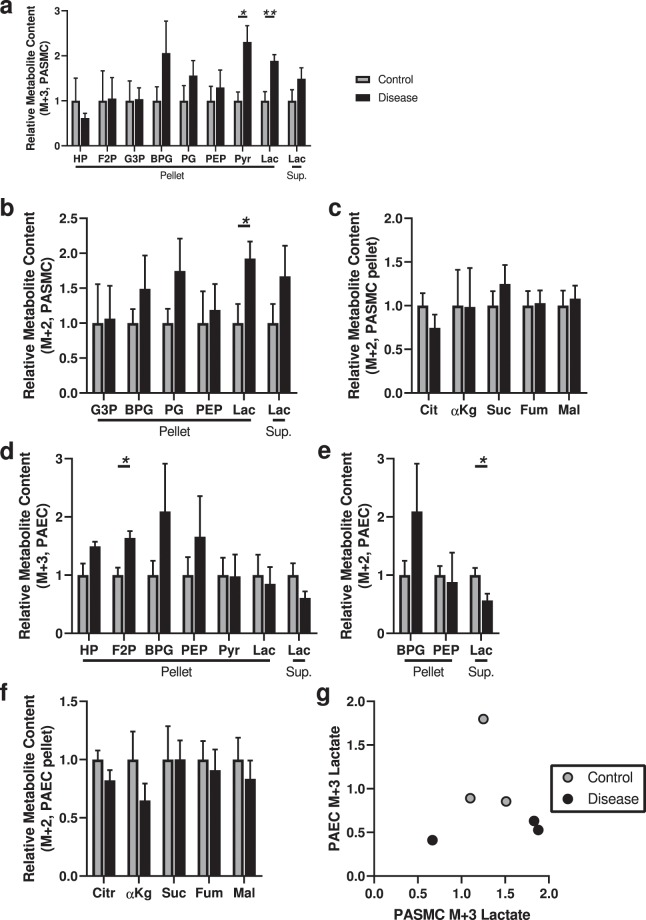
Analysis of ^13^C-labeled metabolite content derived from [1,2,3-^13^C_3_] glucose indicates increased glycolysis and pentose shunt flux in diseased PASMCs, and increased proximate glycolysis metabolites with decreased pentose shunt flux in diseased PAECs. (**a**) ^13^C_3_-Labeled glycolytic intermediates hexose phosphate (HP), fructose bisphosphate (F2P), glyceraldehyde-3-phosphate (G3P), 1,3-bisphosphoglycerate (BPG), phosphoglycerate (PG), phosphoenolpyruvate (PEP), pyruvate (Pyr), and lactate (Lac) in the cell pellet or supernatant (Sup) of control or diseased PASMCs. (**b**) ^13^C_2_-Labeled glycolytic intermediates (derived from pentose shunt metabolism) glyceraldehyde-3-phosphate, 1,3-bisphosphoglycerate, phosphoglycerate, phosphoenolpyruvate, and lactate in in control and diseased PASMCs cell pellet or supernatant; no ^13^C_2_-pyruvate was detected. (**c**) ^13^C-labeled TCA cycle metabolites citrate (Cit), α-ketoglutarate (αKg), succinate (Suc), fumarate (Fum) and malate (Mal); no labeled oxaloacetate was detected. (**d**) ^13^C_3_-Labeled glycolytic intermediates hexose phosphate, fructose bisphosphate, glyceraldehyde-3-phosphate, 1,3-bisphosphoglycerate, phosphoglycerate, phosphoenolpyruvate, pyruvate, and lactate in the cell pellet or supernatant of control or diseased PAECs; no ^13^C_3_-glyceraldehyde-3-phosphate or ^13^C_3_-phosphoglycerate were detected. (**e**) ^13^C_2_-Labeled 1,3-bisphosphoglycerate, phosphoenolpyruvate, and lactate in in control and diseased PAECs cell pellet or supernatant; no ^13^C_2_- glyceraldehyde-3-phosphate, phosphoglycerate, pyruvate or lactate in the pellet was detected. (**f**) ^13^C-labeled citrate, α-ketoglutarate, succinate, fumarate and malate; no labeled oxaloacetate was detected. (N = 5 samples per group, from 5 different control and 5 different disease subjects; unpaired t-test; *P < 0.05, **P < 0.01; mean ± SEM plotted.) (**g**) Scatter plot of M + 3 lactate in the supernatant from PAECs and PASMCs obtained from the same individual (N = 3/group).

We began by assessing the uptake and utilization of [1,2,3-^13^C_3_]glucose in diseased versus control PASMCs. Glucose labeled at carbons 1-3 allows the comparative assessment of glucose metabolism through glycolysis versus the pentose shunt. When metabolized via glycolysis, ^13^C_3_ lactate is generated; in contrast, the activity of pentose shunt enzyme 6-phosphogluconate dehydrogenase evolves ^13^C_1_ as CO_2_, and subsequently glyceraldehyde 3-phosphate and other downstream metabolites re-entering the glycolytic pathway will be labeled as ^13^C_2_^14^. We observed a higher ^13^C-labeled pyruvate and lactate content within diseased PASMCs compared to control PASMCs, although no differences at the level of more proximate metabolites (Fig. 1a): data consistent with the observed shift to glycolysis in PASMCs previously reported^5^. We observed an increase in ^13^C_2_-labeled lactate in the cell pellet similar to that observed with ^13^C_3_ lactate (Fig. 1b), suggestive of increased pentose shunt flux in diseased PASMCs. We also assessed for the ^13^C enrichment of TCA cycle metabolites, but found no significant differences in the diseased cells (Fig. 1c). Overall, these results indicate IPAH PASMCs have increased glucose metabolism through glycolysis and the pentose shunt, but not into the TCA cycle.

### Diseased PAECs have increased proximate glycolysis metabolites, with less pentose shunt metabolism

In the PAECs, we observed a higher content of the proximate glycolysis metabolites, significantly for fructose bisphosphate and a trend for hexose phosphate (*P* = 0.051; hexose phosphate is the combination of the isotopomers glucose phosphate and fructose phosphate), but no differences in more distal metabolites in the glycolytic pathway such as pyruvate or lactate (Fig. 1d). Also in contrast to the PASMCs, we found a reduction in ^13^C_2_-lactate in the supernatant indicative of less pentose shunt flux (Fig. 1e). We found no significant changes in the incorporation of ^13^C-labeled carbons into the TCA cycle in IPAH samples (Fig. 1f).

We looked for correlations in the glycolysis phenotype between PAECs and PASMCs in the cells in each group derived from the same individual (N = 3/group) by assessing lactate in the supernatant, but did not observe any significant correlations (Fig. 1g). We compared cells derived from male versus female PAH subjects, and by pre-transplant PAH medication regimen, but found no differences (Supplementary Figs. 1 and 2). We also looked for correlations with the severity of disease, as measured by mean pulmonary artery pressure pre-transplant, but found no significant correlations (Supplementary Fig. 3). Finally, we assessed BMPR2 mRNA expression by RT-PCR in a subset of the samples (not including 1 sample with a BMPR2 mutation), and looked for correlations in the glycolysis phenotype with BMPR2 expression: we found suggestive inverse correlations in pyruvate content with BMPR2 expression in the cells, but otherwise observed no significant correlations (Supplementary Fig. 4).

### PAECs but not PASMCs have increased glutamine-derived anaplerosis

**Figure 2:**
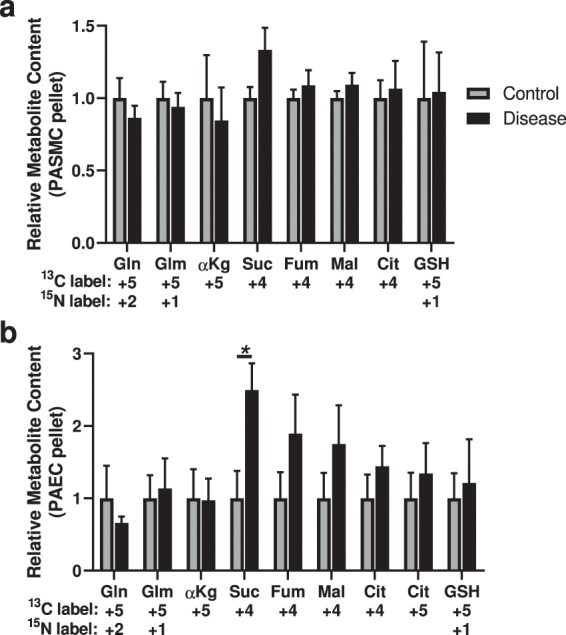
Analysis of ^13^C-labeled metabolite content derived from [^13^C_5_,^15^N_2_] glutamine indicates increased glutamine-derived anaplerosis in diseased PAECs but not PASMCs. (**a**) ^13^C and ^15^N-labeled metabolites glutamine (Gln) and glutamate (Glm); citric acid cycle metabolites α-ketoglutarate (αKg), succinate (Suc), fumarate (Fum), malate (Mal) and citrate (Cit; no ^13^C_4_-oxaloacetate was detected); and glutathione (GSH) in the cell pellet from control and diseased PASMCs. (**b**) ^13^C and ^15^N-labeled metabolites glutamine and glutamate; citric acid cycle metabolites α-ketoglutarate, succinate, fumarate, malate and citrate (no ^13^C_4_-oxaloacetate was detected); and glutathione in the cell pellet from control and diseased PAECs. (N = 5 samples per group, from 5 different control and 5 different disease subjects; unpaired t-test; *P < 0.05; mean ± SEM plotted).

Glutamine is metabolized by cells as an alternative source of carbons to replenish the TCA cycle, a process termed anaplerosis. Using [^13^C_5_,^15^N_2_]glutamine (all 5 carbons and both nitrogens labeled), we found no changes in the incorporation of glutamine-derived carbons into the TCA cycle in diseased PASMCs compared to control cells (Fig. 2a). In PAECs, we observed the diseased cells had more ^13^C-labeled succinate, consistent with increased glutamine-derived anaplerosis (Fig. 2b).

We also detected ^13^C_5_-citrate in the PAECs (Fig. 2b), indicating reductive carboxylation (or retrograde) metabolism in the TCA cycle, which has been previously described in endothelial cells^15^. There was no change in the degree of reductive carboxylation in IPAH cells. This species was not detected in the PASMCs.

### No Evidence of Significantly Altered Long Chain Fatty Acid Utilization by PASMCs or PAECs

We then assessed long chain fatty acid utilization by PASMCs and PAECs. We employed a mixture of 4 uniformly stable isotope-labeled fatty acids, [^13^C_16_]palmitic acid (16:0), [^13^C_16_]palmitoleic acid (16:1), [^13^C_18_]oleic acid (18:1) and [^13^C_18_]lineoleic acid (18:2). We employed a mixture to avoid bias in possible preferential uptake and utilization of specific fatty acids. We assessed the relative content of the four labeled fatty acid substrates remaining in the media of PASMCs and PAECs after 24 hours, and found all four depleted to a comparable degree (data not shown).

**Figure 3:**
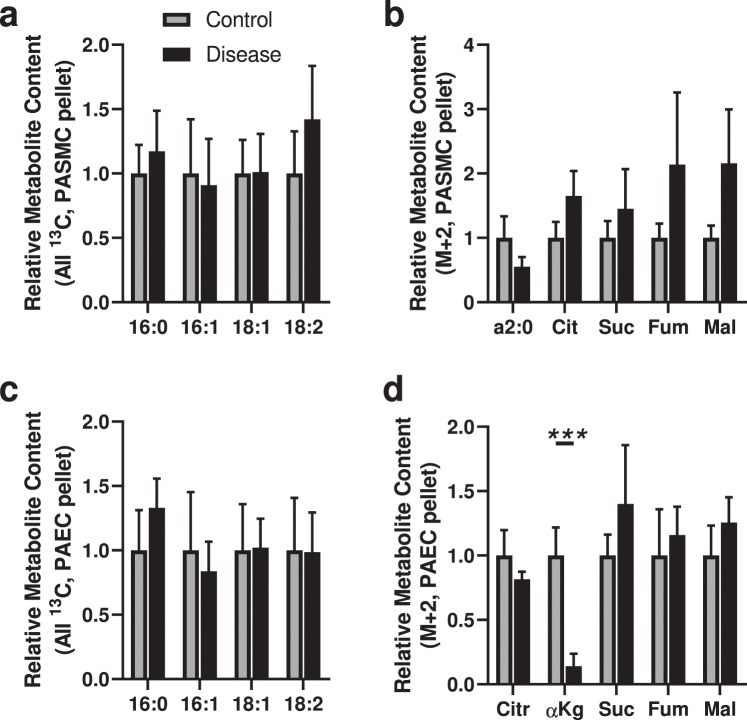
Analysis of ^13^C-labeled metabolite content derived from ^13^C-labeled long chain fatty acids (LCFAs) indicates decreased fatty acid-derived anaplerosis in PAECs but not PASMCs. (**a**) ^13^C-Labeled LCFAs in the cell pellet from control and diseased PASMCs. (**b**) ^13^C_2_-acyl-C2 and ^13^C_2_-labeled citric acid cycle metabolites citrate (Cit), succinate (Suc), fumarate (Fum) and malate (Mal); no ^13^C_2_-α-ketoglutarate or ^13^C_2_-oxaloacetate were detected. (**c**) ^13^C-Labeled LCFAs in the cell pellet from control and diseased PAECs. (**d**) ^13^C_2_-Labeled citric acid cycle metabolites citrate, α-ketoglutarate, succinate, fumarate and malate; no ^13^C_2_-acyl-C2 or ^13^C_2_-oxaloacetate were detected. (N = 5 samples per group, from 5 different control and 5 different disease subjects; unpaired t-test; ***P < 0.005; mean ± SEM plotted).

In PASMCs, we found no differences in the cellular content of the labeled fatty acids between IPAH and control cells (Fig. 3a). We also found no differences in the content of labeled ^13^C-labeled two carbon fragments (the product of β-oxidation breakdown of labeled fatty acids), or ^13^C_2_-citrate resulting from incorporation of the two carbon fragments into the TCA cycle in the diseased cells (Fig. 3b).

In PAECs, we similarly found no significant changes in ^13^C-labeled fatty acid incorporation into the cells (Fig. 3c). There was, however, a decrease in ^13^C enrichment into the TCA cycle at the level of α-ketoglutarate in diseased compared to control cells (Fig. 3d), indicating a decrease in utilization of fatty-acid derived carbons in the TCA cycle.

### TGF-β treatment also promotes increased glycolysis in PASMCs

Increased TGF-β signaling is a key component of many forms of human and experimental PH. We treated PASMCs and PAECs with TGF-β to see if this would phenocopy the disease phenotype in control cells, or augment the phenotype in diseased cells. We used 1 ng/mL of activated TGF-β1, a physiologic dose that has been used extensively in both PASMC^16,17^ and PAEC^18,19^ studies.

**Figure 4:**
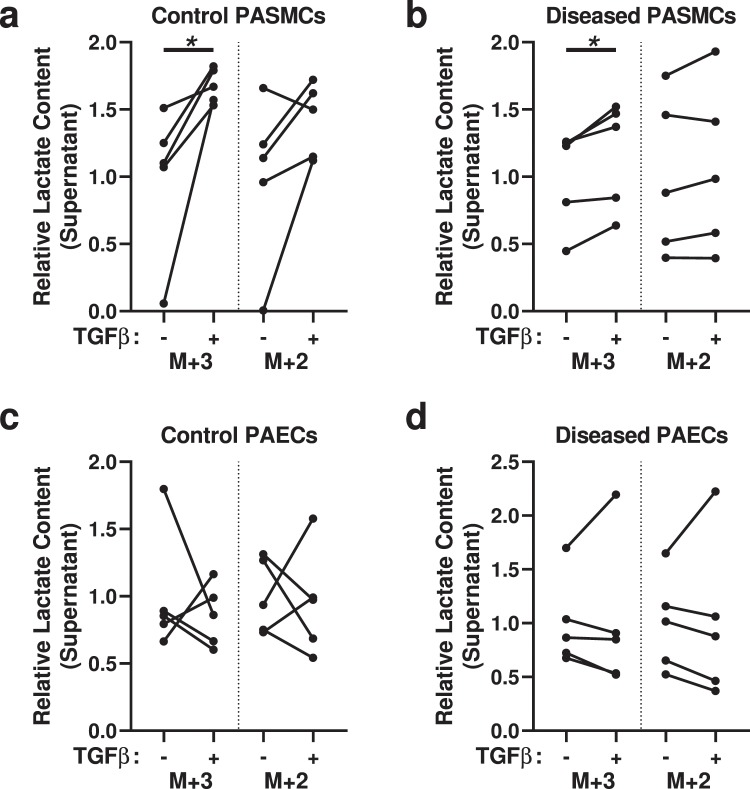
Analysis of ^13^C-labeled metabolite content derived from [1,2,3-^13^C_3_]glucose indicates TGF-β increases glycolysis in PASMCs. (**a**,**b**) ^13^C_3_- and ^13^C_2_-Labeled lactate (M + 3 lactate is derived from glycolysis and M + 2 lactate is derived from pentose shunt metabolism) in the supernatant of (**a**) control or (**b**) diseased PASMCs, treated with or without TGF-β. (**c**,**d**) ^13^C_3_- and ^13^C_2_-Labeled lactate in the supernatant of (**c**) control or (**d**) diseased PAECs, treated with or without TGF-β. (N = 5 samples per group, from 5 different control and 5 different disease subjects; paired t-test; *P < 0.05).

Using the [1,2,3-^13^C_3_]glucose substrate, we observed increased ^13^C_3_ lactate in the supernatant of both control and diseased PASMCs treated with TGF-β, compared to respective untreated cells (Fig. 4a,b), indicating that TGF-β can phenocopy the glycolytic phenotype. We did not, however, find any significant changes in the production of ^13^C_2_ lactate, indicating that TGF-β has less impact on pentose shunt flux. In PAECs, we found TGF-β treatment had no consistent effects on either ^13^C_3_ or ^13^C_2_ labeled glycolysis intermediates or lactate (Fig. 4c,d).

**Figure 5:**
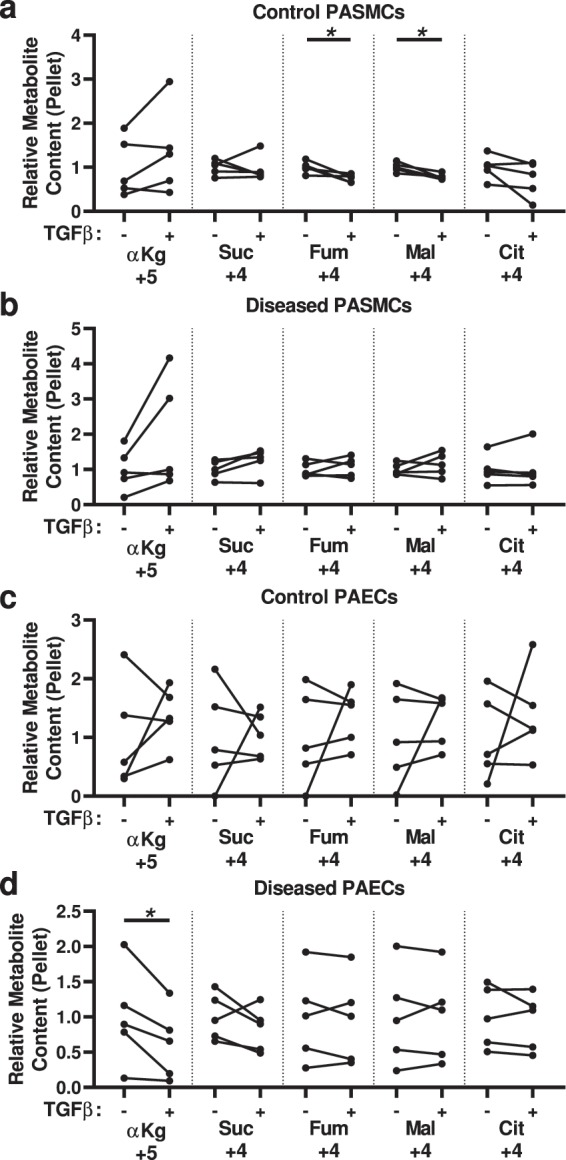
Analysis of ^13^C-labeled metabolites derived from [^13^C_5_,^15^N_2_]glutamine indicates decreased glutamine-derived anaplerosis in control PASMCs treated with TGF-β. (**a**,**b**) ^13^C-Labeled citric acid cycle metabolites α-ketoglutarate (αKg), succinate (Suc), fumarate (Fum), malate (Mal) and citrate (Cit; no ^13^C_4_-oxaloacetate was detected) in the cell pellet from (**a**) control or (**b**) diseased PASMCs, treated with or without TGF-β. (**c**,**d**) ^13^C-Labeled citric acid cycle metabolites α-ketoglutarate, succinate, fumarate, malate and citrate (no ^13^C_4_-oxaloacetate was detected) in the cell pellet from (**c**) control or (**d**) diseased PAECs, treated with or without TGF-β. (N = 5 samples per group, from 5 different control and 5 different disease subjects; paired t-test; *P < 0.05).

Using the [^13^C_5_, ^15^N_2_]glutamine substrate, we found TGF-β treatment of control PASMCs decreased the incorporation of ^13^C carbons into the TCA cycle, at the level of fumarate and malate (Fig. 5a)–in contrast to the absence of a phenotype between diseased and control PASMCs observed above. There was no significant impact of TGF-β on diseased PASMCs (Fig. 5b). TGF-β treatment did not appear to have a consistent impact on glutamine uptake or utilization by PAECs, although TGF-β did decrease ^13^C_5_-labeled α-ketoglutarate in the diseased cells (Fig. 5c,d).

**Figure 6:**
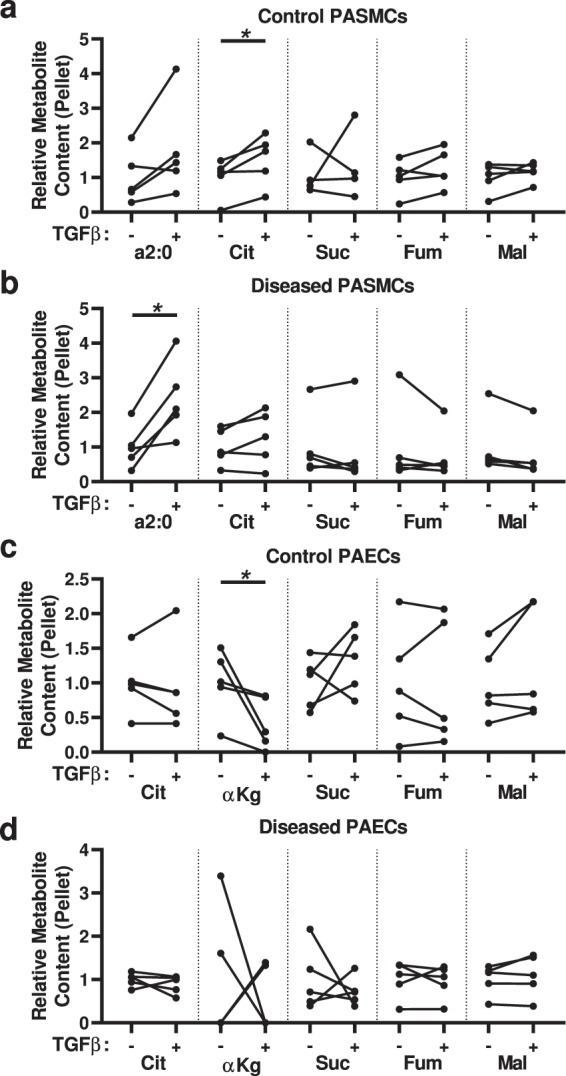
Analysis of ^13^C-labeled metabolites derived from ^13^C-labeled long chain fatty acids (LCFAs) indicates TGF-β increases the incorporation of fatty acid-derived carbons into the TCA cycle in PASMCs but decreases it in PAECs. (**a**,**b**) ^13^C_2_-acyl-C2 (a2:0) and ^13^C_2_-labeled citric acid cycle metabolites citrate (Cit), succinate (Suc), fumarate (Fum) and malate (Mal; no ^13^C_2_-α-ketoglutarate or ^13^C_2_-oxaloacetate were detected) in the cell pellet from (**a**) control or (**b**) diseased PASMCs, treated with or without TGF-β. (**c**,**d**) ^13^C-Labeled citric acid cycle metabolites citrate, α-ketoglutarate (αKg), succinate, fumarate, and malate (no ^13^C_2_-acyl-C2 or ^13^C_2_-oxaloacetate was detected) in the cell pellet from (**c**) control or (**d**) diseased PAECs, treated with or without TGF-β. (N = 5 samples per group, from 5 different control and 5 different disease subjects; paired t-test; *P < 0.05).

Using the U-^13^C-labeled LCFA mixture, we observed control PASMCs treated with TGF-β had an increase in ^13^C_2_ incorporation into citrate (Fig. 6a), and diseased PASMCs treated with TGF-β had an increase in the content of ^13^C-labeled 2 carbon fragments in the cells (Fig. 6b)—indicative of increased fatty acid metabolism. In contrast, TGF-β treatment of control PAECs phenocopied the disease phenotype, with decreased ^13^C_2_-α-ketoglutarate (Fig. 6c,d).

### In TGF-β-treated PASMCs, glucose, glutamine and fatty acids are all carbon sources for the TCA cycle

The TCA cycle is the hub for carbons metabolized by cells, both in terms of a destination for substrate catabolism and a source for anabolism. DCA is thought to function by increasing glucose flux into the TCA cycle, away from lactate^5^. We thus wondered if the metabolic shift seen in TGF-β-treated PASMCs would cause the majority of TCA cycle carbons to be derived from sources other than glucose. To assess this possibility, we sought to determine the relative contribution of carbons to TCA cycle intermediates in TGF-β treated PASMCs. To do so, we used cultured human PASMCs (Lonza) treated with inhibitors of glucose metabolism, glutamine metabolism, or fatty acid metabolism, and then assessed the change in uptake and incorporation of the substrates for each pathway. TGF-β treatment of the cultured PASMCs induced similar metabolic shifts compared to TGF-β treatment of the control donor PASMCs used above, including increased glycolysis and decreased glutaminolysis (data not shown).

**Figure 7:**
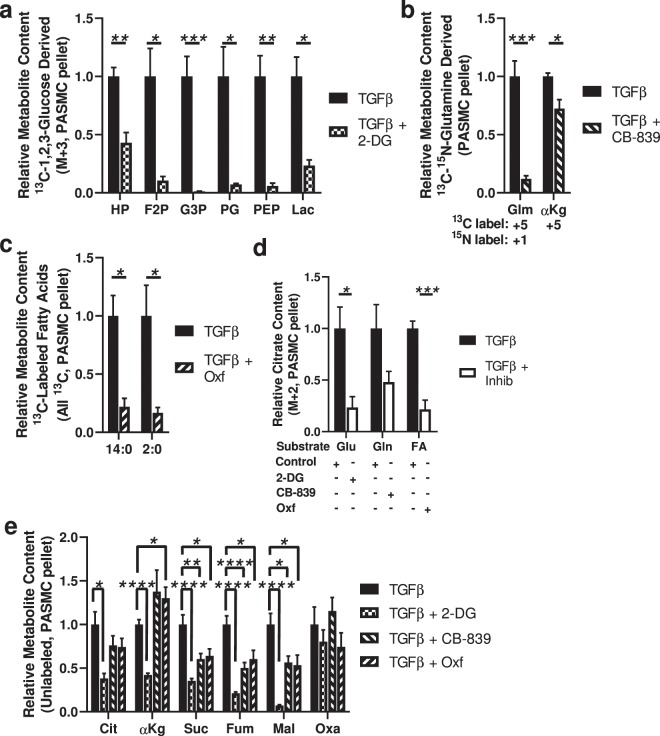
The TCA cycle of TGF-β-treated PASMCs derives carbons from glucose, glutamine and fatty acids. (**a**) Effect of the hexokinase inhibitor 2-deoxyglucose (2-DG) on ^13^C_3_-labeled glycolysis intermediates hexose phosphate (HP), fructose bisphosphate (F2P), glyceraldehyde-3-phosphate (G3P), phosphoglycerate (PG), phosphoenolpyruvate (PEP), and lactate (Lac) derived from [1,2,3-^13^C_3_]glucose in the cell pellet; no labeled ^13^C_3_-1,3-bisphosphoglycerate or ^13^C_3_-pyruvate were detected. (**b**) Effect of the transglutaminase inhibitor CB-839 on ^13^C_5_,^15^N-glutamate (Glm) and ^13^C_5_-α-ketoglutarate (αKg) derived from [^13^C_5_,^15^N_2_]glutamine. (**c**) Effect of the carnitine palmitoyltransferase 1 inhibitor oxfenicine (Oxf) on intracellular fatty acid metabolites derived from ^13^C-labeled fatty acids. (**d**) Effect of 2-deoxyglucose, CB-839 and oxfenicine on the contribution of ^13^C_2_ to citrate deriving from ^13^C-labeled glucose, glutamine and fatty acids, respectively. (**e**) Effect of 2-deoxyglucose, CB-839 and oxfenicine on the total content of the TCA metabolites citrate (Cit), α-ketoglutarate (αKg), succinate (Suc), fumarate (Fum), malate (Mal) and oxaloacetate (Oxa). (A-D: N = 3 samples per group, E: N = 3 samples per group, with 3 replicates, pooled; unpaired t-test between samples; *P < 0.05, **P < 0.01, ***P < 0.005, ****P < 0.001; mean ± SEM plotted).

We first confirmed that the metabolic inhibitors were effective. We observed that inhibiting hexokinase with 2-deoxyglucose significantly blocked the downstream metabolism of glucose (Fig. 7a); inhibiting glutaminase with CB-839 significantly blocked the downstream metabolism of glutamine (Fig. 7b); and inhibiting fatty acid transport into the mitochondria with the carnitine palmitoyltransferase 1 (CPT1) inhibitor oxfenicine significantly blocked fatty acid β-oxidation (Fig. 7c).

We then quantified the relative incorporation of labeled substrates into ^13^C_2_-citrate with and without inhibition. We found that there were significant decreases in the incorporation of glucose blocked by 2-deoxyglucose and fatty acids blocked by oxfenicine, and a modest trend (*P* = 0.11) towards less incorporation of glutamine after CB-839 treatment (Fig. 7d). We then quantified the total (unlabeled) content of TCA cycle intermediates. We found significant decreases in the content of multiple TCA metabolites intermediates with all 3 treatments–2-deoxyglucose, CB-839, and oxfenicine–with the most significant decreases observed with 2-deoxyglucose treatment (Fig. 7e). These data indicate that in TGF-β-treated PASMCs–despite metabolic shifts including increased glycolysis, decreased glutamine-derived anaplerosis, and increased fatty acid β-oxidation–glucose, glutamine and fatty acids all contribute carbons to the TCA cycle, although potentially glucose more so than the others.

## Discussion

Shifts in cellular metabolism have long been recognized as a hallmark of cancer pathobiology^20^, and are increasingly recognized in PH as well. In both cancer and PH, one of the critical metabolic shifts is increased aerobic glycolysis (also known as the Warburg phenomenon), which supports a pro-proliferative, anti-apoptotic phenotype^1^. In addition to increased glucose metabolism to lactate and not to TCA cycle intermediates, the Warburg phenomenon also includes aberrant mitochondrial oxygen sensing through epigenetic mechanisms, effectively resulting in “a state of pseudohypoxia” which contributes to PH pathogenesis^21^. Molecularly, hypoxia-inducible factor-1α is a master regulator of glycolysis, and is increased in both PH and cancer^22–24^.

Compared to glycolysis, pentose shunt metabolism has been less studied in PH. The enzyme which regulates glucose-6-phosphate entry into the pentose shunt, glucose-6-phosphate dehydrogenase, has been observed to be increased in hypoxic PASMCs^25^. One function of the pentose pathway is the generation of cytosolic NADPH, which can be used to reduce oxidized glutathione (GSSG) and regenerate active site thiols in antioxidant enzymes in settings of oxidative stress which may be present in PH.

Altered TGF-β family signaling underlies many forms of pulmonary hypertension (PH), as evidenced by mutations in TGF-β pathway members underlying heritable PH; increased TGF-β signaling present in human and animal PH model lung tissue; and excessive activation of latent TGF-β^26^. Blockade of TGF-β signaling is protective in multiple forms of experimental PH^13,27–29^, and clinical trials targeting TGF-β family ligands and receptors are currently ongoing. However, mechanisms by which aberrant TGF-β signaling results in the vascular pathology have not been fully elucidated. Knockdown of BMPR2 has shown to promote glycolysis^30^; here in small numbers of samples we did not observe consistent trends between BMPR2 expression and glycolysis parameters. We did find evidence that TGF-β treatment can promote and augment a shift to glycolysis in PASMCs. These results are consistent with data that TGF-β can increase glucose utilization by fibroblasts^31^, although TGF-β did not phenocopy all aspects of the metabolic phenotype in IPAH cells.

We used stable isotope metabolic tracing *in vitro* in conjunction with mass spectrometry-based metabolomics to evaluate the uptake and utilization of three key metabolic substrates: glucose, glutamine and fatty acids. ^13^Carbon and ^15^N are non-radioactive and do not decay, and thus can be used for the tracing of labeled atoms into downstream metabolites^32^. Enzymes have a mild substrate preference for molecules containing lighter isotopes, which may induce bias in ^13^C-tracking studies, but in general these effects are thought to be very minor^33^. Substrates other than glucose, glutamine and fatty acids may serve as alternative carbon and energy sources in mammalian cells, but these three compounds account for the majority of substrates. In the context of pathology, cells shift how carbon atoms flow through the cells: for example, in cancer cells the shift in glucose utilization to glycolysis from glucose oxidation is accompanied by increased glutaminolysis to fuel the TCA cycle^34^. All three substrates have the potential to contribute carbon atoms to the TCA cycle, as we observed with PASMCs here.

Stable isotope metabolomics complements steady state metabolomics, which assesses the content of unlabeled metabolites in cells. Fessel *et al*. used steady-state metabolomics to study human PAECs transfected with mutant bone morphogenetic protein receptor 2 (BMPR2), a TGF-β receptor family member and a common driver of heritable human PH^35^. The authors found an increase in proximate glycolytic pathway intermediates, which corresponds well with our data finding an increase in proximate ^13^C-labeled glycolytic pathway intermediates derived from ^13^C-labeled glucose. They also observed a decrease in fatty acid β-oxidation intermediates, which corresponds to the decrease in ^13^C-labeled α-ketoglutarate derived from ^13^C-fatty acid catabolism we observed. Finally, they also reported a decrease in glutamine-derived metabolites; here, our results diverge as we found evidence of increased labeled succinate derived from glutamine.

Endothelial cell proliferation has been reported to require all three of glycolysis, fatty acid β-oxidation and glutamine metabolism. In endothelial cells, glycolytic flux is mediated by upregulation of PFKFB3, and blockade of PFKFB3 can suppress angiogenesis^36^. Fatty acid metabolism is also required for angiogenesis, but here the carbons are used for *de novo* nucleotide synthesis to support DNA replication^37^. Blocking glutamine metabolism induces endothelial cell senescence^38^; as observed by ourselves and others^15^, glutamine-derived α-ketoglutarate can undergo reductive carboxylation, likely to support the fatty acid and phospholipid anabolism required to maintain membrane homeostasis in these cells with a very high ratio of surface area to volume^39^. The requirement for membrane synthesis is PAECs also supported by our observation that ^13^C labeled carbons may leave glycolysis at an intermediate step: glycerol is produced from glyceraldehyde 3-phosphate, and is also a critical component of phospholipids.

In PH, diseased PAECs have a decrease in oxygen consumption (corresponding to a decrease in mitochondrial respiration), and the density of mitochondria per cell is decreased in IPAH-derived PAECs compared to control PAECs^6^. Increased stiffness of the underlying matrix is sufficient to induce increased glycolysis and YAP/TAZ-dependent glutaminolysis in PAECs, a metabolic shift which supports proliferation of the cells^10^, and which we also found evidence for here in IPAH-derived cells. Endothelial-to-mesenchymal transition (EndoMT) of ECs, induced *in vitro* by a combination of TGF-β and IL-1β, causes a decrease in fatty acid β-oxidation, while suppressing fatty acid β-oxidation (as we observed occurred in the PAECs treated with TGF-β) is sufficient to induce EndoMT^40^.

To analyze fatty acid metabolism here we used a stable isotope-labeled mixture of palmitic acid, palmitoleic acid, oleic acid and lineolic acid to assess uptake and utilization in PASMCs and PAECs. This approach has the disadvantage of not allowing the discrete analysis of specific fatty acid substrates, which could be handled differently. However, the relevance of the substrates we analyzed is supported by the reported increase in circulating plasma concentrations of palmitic acid, oleic acid and lineolic acid by 12.0, 5.0 and 4.9-fold, respectively, in PAH versus control subjects^41^.

The pathologic phenotype of PH cells can be reversed, at least in part, by targeting the altered metabolism. Excessive proliferation and apoptosis resistance in PASMCs can be reversed by increasing glucose oxidation by treating the cells with DCA or blocking fatty acid oxidation such as with ranolazine, which increases glucose intake into the cells via the Randle cycle^5,41,42^. Our studies broaden these data, with the observation that even in the context of increased glycolysis induced by TGF-β in PASMCs, glucose remains a major source of carbons for the TCA cycle.

The possibility of targeting the PH pathology by modulating metabolism has led to clinical trials using DCA as a pharmacologic treatment for PAH, which has shown promise particularly in individuals who lack genetic mutations in other pathways^9^. If pharmacologic TGF-β modulation has clinical benefit, such as by the ligand trap soterdacept, it is likely that a beneficial mechanism will be through modulating cellular metabolism. By targeting similar pathways, metabolic treatments may be found to have a synergistic effect with TGF-β pathway modulators.

An important limitation on making comparisons between PASMCs and PAECs in our study is the use of different cell culture media specific for smooth muscle and endothelial cells, which likely impacts the metabolic phenotype of the cells. For example, the smooth muscle cell media we used contains 2% fetal bovine serum (FBS) whereas the endothelial cell media contains 5% FBS: FBS contains hormones and other growth factors such as insulin and cortisol that impact metabolism. Another limitation on comparing the relative uptake between different labeled metabolites is that the stable isotope-labeled metabolites were added to the media, adding to unlabeled metabolites already present. For example, the addition of [1,2,3-^13^C_3_]glucose increased the total glucose concentration in the media, which may itself increase glucose uptake by cells^42^. We also have not, here, directly identified which metabolic pathways contribute to specific pathologic phenotypes in each cell type. Other limitations include the relatively small sample size, as primary cell lines obtained from cultured organs are a precious and scarce resource, and related to this we used cells at relatively high passage numbers because of the large number of experiments and numbers of cells required to generate high fidelity results.

In summary, we found evidence of a metabolic shift in diseased PASMCs, including increased glycolysis and pentose shunt flux. In PAECs, we found more modest changes, including an increase in several proximate intermediates in glycolysis, less pentose shunt flux, and increased glutamine-derived anaplerosis with less fatty acid fueling of the TCA cycle. These data suggest that therapeutic strategies addressing the altered cellular metabolism in PH may be most effective if they target specific cell compartments.

## Methods

### Cells and culture techniques

#### Primary PASMCs and PAECs

Primary PASMCs and PAECs were obtained from the cell studies core of the Pulmonary Hypertension Breakthrough Initiative (PHBI) Research Network (Philadelphia, PA). The PASMCs were cultured in Smooth Muscle Cell Medium (SMCM; ScienCell Research Laboratories, Carlsbad, CA; Cat#1101). The PAECs were cultured in Vasculife EC complete kit (Lifeline Cell Technology, Frederick, MD; Cat#LL-0005). Both the PASMC and PAEC media contain 5.5 mM glucose and 10 mM glutamine. Both PASMCs and PAECs were cultured at 37 °C with an atmosphere of 5% CO_2_ (95% air) and 100% humidity. The cells were grown to no more than 90% confluence, and sub-cultured at a density of a least 5.0 × 10^5^ cells in 100 mm diameter dishes. Both primary cell types were used between passages 7 and 9. Viability was determined using trypan blue exclusion dye by light microscopy.

#### Cultured PASMCs

For the metabolic inhibitor experiments, we used cultured PASMCs from Lonza (Walkersville, MD; Cat#CC-2581). The cells were cultured as described above in SMCM.

#### Sample collection

PASMCs and PAECs from 5 controls (Failed Donors) and 5 diseased subjects (idiopathic pulmonary arterial hypertension; IPAH; see Table 1) were cultured in their respective culture media to 85–90% confluence, adding fresh media every 2–3 days (the rate of growth varied depending on cell type). When the cultures reached target confluence, the cells were treated under different conditions for 24 hours, at 37 °C with an atmosphere of 5% CO_2_ (95% air) and 100% humidity. After 24 hours treatment, the cells were harvested; collecting 1 ml of supernatant media which was snap frozen in liquid nitrogen. The rest of the media was removed from the plate and the cells were washed twice with sterile PBS and trypsinized for 2–5 min at 37 °C. 3 ml of the respective cold media was added and the plate washed with 2 ml of cold ice PBS. The cells were pooled in 15 ml conical tubes and centrifuged 5 min at 1000 rpm at room temperature. The recovered pellet was then resuspended into 1 ml of ice cold PBS, and an aliquot counted via heamocytometer. The cell suspension was centrifuged 3 min at 1600rpm at 4 °C and the final pellet snap frozen in liquid N_2_. Media supernatants and cell pellets were stored at −70 °C until mass spectrometry analysis. Experiments using cultured PASMCs were performed in the same manner.

#### BMPR2 quantification

mRNA was isolated from PASMCs and PAECs, and RT-PCR used to quantify expression of BMPR2 and β-actin using the 2^−ΔCt^ method.

### Treatments, including labeled metabolic substrates

Primary PASMCs and PAECs were treated with or without 1 ng/ml human recombinant human TGF-β1 (Invitrogen, Carlsbad, CA; Cat# 14-8348-62) at the same time as metabolic substrates were added to the respective culture media for the same duration of time, 24 hours. The metabolic substrates were 1 mg/ml of [1,2,3-^13^C_3_]glucose (Sigma-Aldrich, St. Louis, MO, Cat#720127); 4 mM [^13^C_5_;^15^N_2_]glutamine (Cambridge Isotope Laboratories Inc., Tewksbury, MA; Cat# CNLM-1275-H); and 100 µM ^13^C_U_-mixed fatty acids (Cambridge Isotope Laboratories Inc.; Cat# CLM-8455-1).

Cultured PASMCs were used for inhibition experiments using the same protocols as above, with the further addition of metabolic inhibitors, with 10 µM CB-839 (Sellechem, Houston TX; Cat# S7655); 1 mM 4-hydroxy-L-phenylglycine (oxfencine; Sigma-Aldrich; Cat# 56160); or 2 mg/ml 2-deoxy-D-glucose (Sigma-Aldrich; Cat# D8375).

### Metabolomics assessment

Frozen cell pellets were extracted at 2 × 10^6^ cells/mL in ice cold lysis/extraction buffer (methanol:acetonitrile:water 5:3:2, v/v/v). Twenty µL of supernatant samples was extracted with 480 µL of extraction buffer. Samples were agitated at 4 °C for 30 min followed by centrifugation at 10,000 g for 10 min at 4 °C. Protein and lipid pellets were discarded, and extracts were injected (10 µL for cell samples; 20 µL for supernatant samples) into a UHPLC system (Vanquish, Thermo, San Jose, CA, USA) and run on a Kinetex C18 column (150 × 2.1 mm, 1.7 µm – Phenomenex, Torrance, CA, USA). Solvents were Optima H_2_O (Phase A) and Optima acetonitrile (Phase B) supplemented with 0.1% formic acid for positive mode runs and 1 mM NH_4_OAc for negative mode runs. Samples were analyzed using a 9 min gradient from 5–95% organic phase at 400 µL/min^43^. The autosampler was held at 7 °C for all runs; the column compartment was held at 45 °C. The UHPLC system was coupled online with a Q Exactive mass spectrometer (Thermo, Bremen, Germany), scanning in Full MS mode (2 µscans) at 70,000 resolution in the 60–900 m/z range in negative and then positive ion mode (separate runs). Eluate was subjected to electrospray ionization (ESI) with 4 kV spray voltage. Nitrogen gas settings were 45 sheath gas and 15 auxiliary gas. Metabolite assignments and isotopologue distributions were determined using Maven (Princeton, NJ, USA), following conversion of.raw files to.mzXML format through RawConverter. Chromatographic and MS technical stability were assessed by determining CVs for heavy and light isotopologues in a technical mixture of extract run every 10 injections.

### Statistics

Relative quantitation was performed by exporting the values for integrated peak areas of light metabolites and their isotopologues into Excel (Microsoft, Redmond, CA, USA) for statistical analysis including t-test and ANOVA (significance threshold for p-values < 0.05). Data was plotted using GraphPad Prism (La Jolla, CA, USA). *P* < 0.05 was considered statistically significant.

## Supplementary information

Supplementary File 1.

## Data Availability

The datasets generated during and/or analyzed during the current study are available from the corresponding author on reasonable request.
