# Publisher Correction: Stable isotope metabolomics of pulmonary artery smooth muscle and endothelial cells in pulmonary hypertension and with TGF-beta treatment

**DOI:** 10.1038/s41598-020-60500-w

**Published:** 2020-03-04

**Authors:** Daniel Hernandez-Saavedra, Linda Sanders, Scott Freeman, Julie A. Reisz, Michael H. Lee, Claudia Mickael, Rahul Kumar, Biruk Kassa, Sue Gu, Angelo D’ Alessandro, Kurt R. Stenmark, Rubin M. Tuder, Brian B. Graham

**Affiliations:** 1 Department of Medicine, University of Colorado Anschutz Medical Campus, Aurora, CO USA; 2 Department of Biochemistry and Molecular Genetics, University of Colorado Anschutz Medical Campus, Aurora, CO USA; 3 Department of Medicine, University of California San Francisco, San Francisco, CA USA; 4 Department of Pediatrics, University of Colorado Anschutz Medical Campus, Aurora, CO USA

Correction to: *Scientific Reports* 10.1038/s41598-019-57200-5, published online 15 January 2020

In the original version of this Article, the author Angelo D’ Alessandro was incorrectly indexed. This error has now been corrected.

