# Supplementary material for: Stable isotope metabolomics of pulmonary artery smooth muscle and endothelial cells in pulmonary hypertension and with TGF-beta treatment
Source: Sci Rep. 2020 Jan 15;10:413. doi: 10.1038/s41598-019-57200-5 (PMC6962446; doi:10.1038/s41598-019-57200-5)
Supplement: Supplementary file 1 [file 41598_2019_57200_MOESM1_ESM.docx]

**Supplementary Figures**

Stable isotope metabolomics of pulmonary artery smooth muscle and endothelial cells in pulmonary hypertension and with TGF-beta treatment

Daniel Hernandez-Saavedra, Linda Sanders, Scott Freeman, Julie A. Reisz, Michael H. Lee, Claudia Mickael, Rahul Kumar, Biruk Kassa, Sue Gu, Angelo D’Alessandro, Kurt R. Stenmark, Rubin M. Tuder, Brian B. Graham


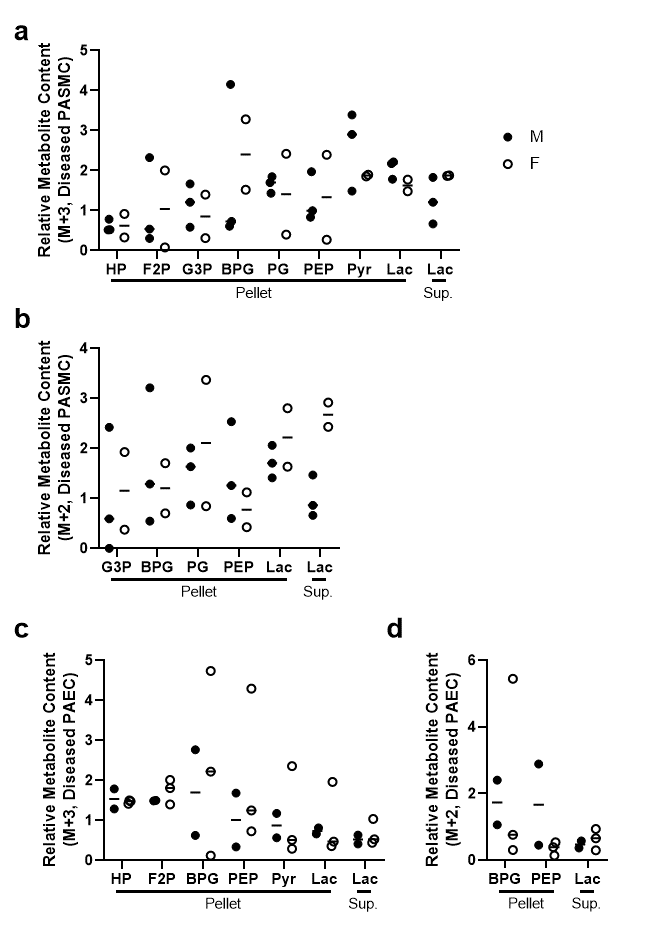


**Supplementary Figure 1. Analysis of ^13^C-labeled metabolite content derived from [1,2,3-^13^C_3_]glucose in diseased PASMCs and PAECs, by male versus female sex.** (**a**) ^13^C_3_-Labeled glycolytic intermediates hexose phosphate (HP), fructose bisphosphate (F2P), glyceraldehyde-3-phosphate (G3P), 1,3-bisphosphoglycerate (BPG), phosphoglycerate (PG), phosphoenolpyruvate (PEP), pyruvate (Pyr), and lactate (Lac) in the cell pellet or supernatant (Sup) of control or diseased PASMCs. (**b**) ^13^C_2_-Labeled glycolytic intermediates (derived from pentose shunt metabolism) glyceraldehyde-3-phosphate, 1,3-bisphosphoglycerate, phosphoglycerate, phosphoenolpyruvate, and lactate in in control and diseased PASMCs cell pellet or supernatant; no ^13^C_2_-pyruvate was detected. (**c**) ^13^C_3_-Labeled glycolytic intermediates hexose phosphate, fructose bisphosphate, glyceraldehyde-3-phosphate, 1,3-bisphosphoglycerate, phosphoglycerate, phosphoenolpyruvate, pyruvate, and lactate in the cell pellet or supernatant of control or diseased PAECs; no ^13^C_3_-glyceraldehyde-3-phosphate or ^13^C_3_-phosphoglycerate were detected. (**d**) ^13^C_2_-Labeled 1,3-bisphosphoglycerate, phosphoenolpyruvate, and lactate in in control and diseased PAECs cell pellet or supernatant; no ^13^C_2_- glyceraldehyde-3-phosphate, phosphoglycerate, pyruvate or lactate in the pellet was detected. (N=2-3 samples per sex per group; median plotted.)


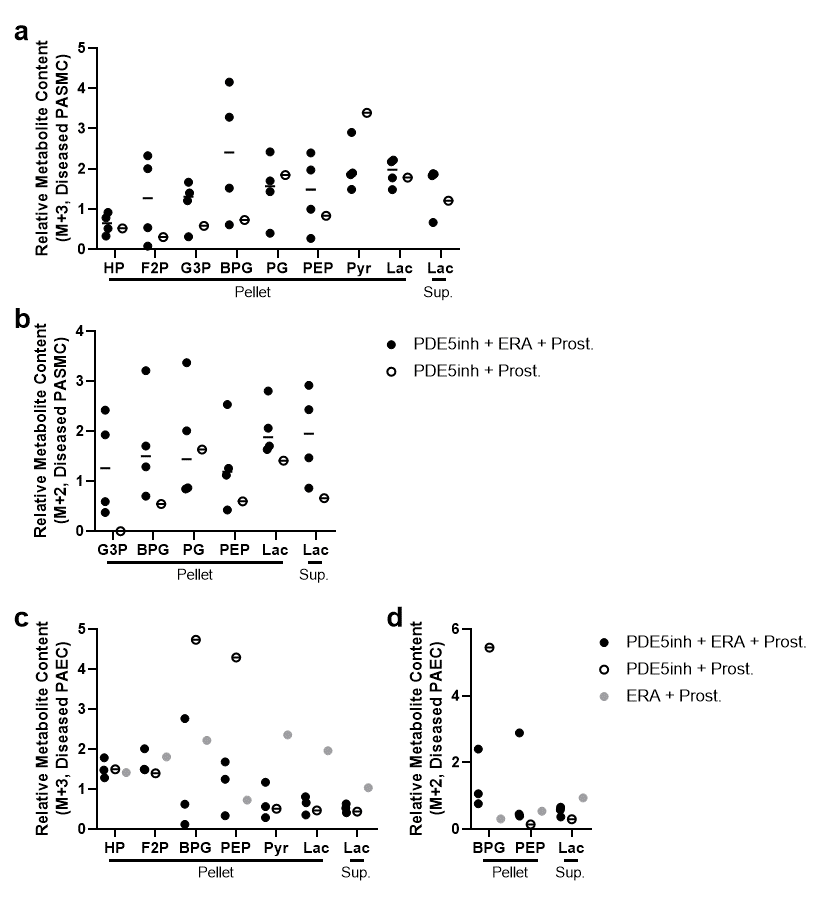


**Supplementary Figure 2. Analysis of ^13^C-labeled metabolite content derived from [1,2,3-^13^C_3_]glucose in diseased PASMCs and PAECs, by pre-transplant treatment regimen.** PDE5inh: PDE5 inhibitor; ERA: endothelin receptor antagonist; Prost.: prostacyclin analog. (**a**) ^13^C_3_-Labeled glycolytic intermediates hexose phosphate (HP), fructose bisphosphate (F2P), glyceraldehyde-3-phosphate (G3P), 1,3-bisphosphoglycerate (BPG), phosphoglycerate (PG), phosphoenolpyruvate (PEP), pyruvate (Pyr), and lactate (Lac) in the cell pellet or supernatant (Sup) of diseased PASMCs. (**b**) ^13^C_2_-Labeled glycolytic intermediates (derived from pentose shunt metabolism) glyceraldehyde-3-phosphate, 1,3-bisphosphoglycerate, phosphoglycerate, phosphoenolpyruvate, and lactate in diseased PASMCs cell pellet or supernatant; no ^13^C_2_-pyruvate was detected. (**c**) ^13^C_3_-Labeled glycolytic intermediates hexose phosphate, fructose bisphosphate, glyceraldehyde-3-phosphate, 1,3-bisphosphoglycerate, phosphoglycerate, phosphoenolpyruvate, pyruvate, and lactate in the cell pellet or supernatant of diseased PAECs; no ^13^C_3_-glyceraldehyde-3-phosphate or ^13^C_3_-phosphoglycerate were detected. (**d**) ^13^C_2_-Labeled 1,3-bisphosphoglycerate, phosphoenolpyruvate, and lactate in diseased PAECs cell pellet or supernatant; no ^13^C_2_- glyceraldehyde-3-phosphate, phosphoglycerate, pyruvate or lactate in the pellet was detected. (N=1-4 samples per treatment per group; median plotted.)


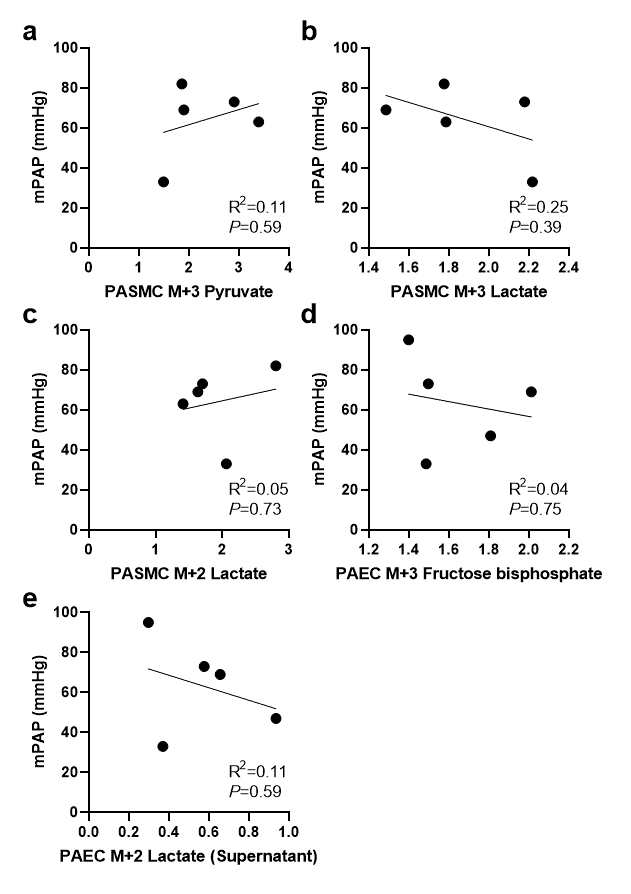


**Supplementary Figure 3. Analysis of ^13^C-labeled metabolite content derived from [1,2,3-^13^C_3_]glucose in diseased PASMCs and PAECs, plotted against mean pulmonary artery pressure (mPAP) prior to transplant.** (**a, b** and **c**) ^13^C_3_- and ^13^C_2_-labeled glycolytic intermediates pyruvate and lactate in the cell pellet of diseased PASMCs, plotted against mPAP. (**d** and **e**) ^13^C_3_-labeled fructose bisphosphate in the cell pellet and ^13^C_2_-labeled lactate in the supernatant of diseased PAECs, plotted against mPAP. (N=5 samples per group; linear regression plotted with calculated R^2^ and *P* values shown.)


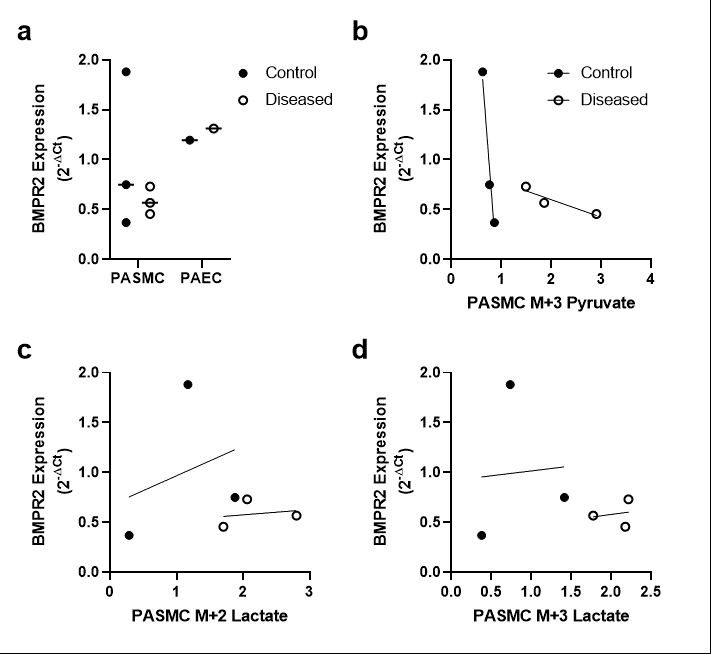


**Supplementary Figure 4. Analysis of BMPR2 expression, and correlations between BMPR2 expression and ^13^C-labeled metabolite content derived from [1,2,3-^13^C_3_]glucose in control and diseased PASMCs.** (**a**) BMPR2 expression by RT-PCR in PASMCs and PAECs (2^-ΔCt^ method, using β-actin as the housekeeping gene, N=1-3 samples per group). (**b, c** and **d**) ^13^C_3_- and ^13^C_2_-labeled glycolytic intermediates pyruvate and lactate in the cell pellet of diseased PASMCs, plotted against BMPR2 expression. (N=3 samples per group; linear regression plotted.)
